# Safety of Cryopreserved Stem Cell Infusion through a Peripherally Inserted Central Venous Catheter

**DOI:** 10.3390/cancers15041338

**Published:** 2023-02-20

**Authors:** Sławomir Milczarek, Piotr Kulig, Alina Zuchmańska, Bartłomiej Baumert, Bogumiła Osękowska, Anna Bielikowicz, Ewa Wilk-Milczarek, Bogusław Machaliński

**Affiliations:** 1 Department of General Pathology, Pomeranian Medical University, 70-111 Szczecin, Poland; 2 Department of Hematology and Transplantology, Pomeranian Medical University, 71-252 Szczecin, Poland; 3 Department of General and Dental Radiology, Pomeranian Medical University, 70-111 Szczecin, Poland

**Keywords:** PICC, CICC, auto-HSCT, allo-HSCT, high-dose chemotherapy, hematological malignancy, non-hematological malignancy

## Abstract

**Simple Summary:**

Autologous and allogeneic hematopoietic stem cell transplantations (HSCTs) are considered the standard of care for many hematological and non-hematological malignancies. It is generally believed that cryopreserved hematopoietic stem cells should be administered rapidly to minimize their exposure to cytotoxic dimethyl sulfoxide (DMSO). A fast peripheral blood stem cell (PBSC) infusion rate is usually achieved through conventionally inserted central venous catheters (CICCs). Therefore, CICCs are widely used in PBSC transplantation settings. Nevertheless, their insertion may be associated with serious complications. Peripherally inserted central venous catheters (PICCs) are not associated with such complications but are rarely used in cryopreserved PBSC transplantation due to the presumption that prolonged PBSC infusion through the CICC would impair their viability. We demonstrated that prolonged PBSC infusion through the PICC has no effect on transplantation outcomes. Therefore, we suggest implementing PICCs in a cryopreserved transplantation setting, as PICCs are more comfortable for patients, easier to insert, cost-effective, and have fewer serious line-related complications.

**Abstract:**

The management of patients undergoing stem cell transplantation requires a multipurpose central venous catheter (CVC) to facilitate drug administration, parenteral nutrition, transfusion of blood products, and collection of blood samples. Peripherally inserted central venous catheters (PICCs) appear to meet these requirements but are rarely used for stem cell infusion. We aimed to retrospectively assess the safety and feasibility of stem cell infusion through PICC and to evaluate its impact on transplantation kinetics. We retrospectively analyzed the outcomes of peripheral blood stem cell (PBSC) transplantation in patients receiving cryopreserved autologous or allogeneic PBSC by PICCs and compared the results with patients receiving transplants through a conventionally inserted central venous catheter (CICC). Despite statistically significant differences in CD34^+^ dose, infusion rate, and total length of administration, the clinical outcomes of transplantation, exemplified by platelet and neutrophil engraftment, along with the length of hospitalization, were not affected by the prolonged infusion time and lower infusion velocity in the PICC group. Our study showed that the clinical outcomes of PBSC transplantation did not differ between the PICC and CICC groups, suggesting that both types of catheters can be implemented in a PBSC transplantation setting.

## 1. Introduction

Autologous and allogeneic hematopoietic stem cell transplantations (HSCTs) are considered the standard of care for many hematological malignancies. Moreover, allogeneic HSCT can be implemented in the treatment of various non-malignant hematological diseases such as bone marrow failure disorders, either acquired or inherited, immune dysregulation disorders, and hemoglobinopathies [1]. Both autologous and allogenic HSCTs are considered the standard of care for multiple hematological malignancies [2]. Currently, HSCTs are a standard of care for newly diagnosed multiple myeloma (NDMM) patients as consolidation therapy. Moreover, it can be considered a salvage therapy in relapsed, refractory multiple myeloma [3]. In addition, recently, the indications for HSCT have expanded beyond hematology. Autologous HSCT is a clinical option in the treatment of relapsed and refractory germ cell tumors and is associated with relatively good clinical outcomes [4]. Interestingly, HSCT is believed to have therapeutic potential in various autoimmune diseases. The rationale for this approach is based on the concept of immunoablation using high-dose chemotherapy followed by the regeneration of naive T cells derived from hematopoietic progenitor cells. Promising results have been reported in the treatment of some gastrointestinal disorders, such as inflammatory bowel disease, refractory celiac disease, and some chronic liver diseases [5]. In addition, HSCTs were implemented in the therapy of systemic sclerosis, systemic lupus erythematosus, and juvenile rheumatoid arthritis [6], as well as in multiple sclerosis [6,7]. Moreover, the application of immunoablation followed by autologous HSCT in amyotrophic lateral sclerosis is under ongoing debate [8].

The management of patients undergoing HSCT requires the insertion of a multipurpose central venous catheter (CVC) for chemotherapy infusion, antibiotics administration, parenteral nutrition, transfusion of blood products, and blood sample collection. In this context, adequate venous access is essential for the uncomplicated management of patients with poor vasculature due to previous cycles of chemotherapy. Typically, the conventionally inserted central venous catheter (CICC) remains the preferred access device in most transplant centers as it is routinely implanted, and its dual or triple lumen facilitates the simultaneous infusion of physically incompatible drugs and blood products. Furthermore, CICC allows for a rapid stem cell infusion rate that is considered optimal.

Cryopreserved peripheral blood stem cell (PBSC) products are typically thawed at the patient’s bedside and immediately infused into the patient by gravity through a large bore venous line. This procedure is believed to minimize exposure to dimethyl sulfoxide (DMSO). Therefore, it prevents the release of potentially harmful cytokines and cell debris that can cause loss of PBSC viability [9,10]. PICCs are rarely used to administer hematopoietic progenitor cells. The explanation for this practice appears not to be based on actual evidence but rather on the assumption that the smaller gauge size of the PICC and the resulting reduced infusion rates would impair PBSC viability, contributing to poor graft function or engraftment failure. It is worth mentioning that the use of PICCs has increased significantly in the last few years because they improve patient comfort, are easier to insert, are cost-effective, and have fewer serious line-related complications [11,12,13].

Here, we would like to present the results of our retrospective observational study on the use of PICCs in patients receiving cryopreserved stem cells. Our aim was to assess the safety and feasibility of prolonged infusion by PICC and to evaluate its impact on engraftment kinetics. We hypothesized that prolonged infusions of cryopreserved stem cells have minimal effects on platelet and neutrophil engraftment and the overall length of hospitalization in stem-cell transplant recipients. 

## 2. Materials and Methods

### 2.1. Patients and Catheters Characteristics

From January 2020 to October 2022, a retrospective observational study was conducted at the Independent Public Hospital No. 1 of the Pomeranian Medical University in Szczecin (Poland). A total of 49 patients with malignant diseases undergoing autologous or allogeneic PBSC transplantation were enrolled in the study. Each patient received previously cryopreserved stem cells that were thawed at the patient’s bedside. All patients suffered from hematological malignancies or solid tumors: plasma cell myeloma (PCM, n = 44), light chain amyloidosis (AL, n = 1), acute myeloid leukemia (AML, n = 1), and germ cell tumors (GCT, n = 3). One PCM patient underwent tandem autologous stem cell transplantation (ASCT), and two GCT-affected patients underwent triple ASCT. Patient characteristics are presented in Table 1.

The patients were divided into 2 groups—the first group consisted of 16 patients who received transplantation by PICC, while the second/control group consisted of 33 patients who received stem cells by CICC with large bore. Catheters were inserted up to 3 days before the initiation of the conditioning regimen, except for 2 patients with GCT who underwent 2 transplantations using a single PICC. At our center, catheters are implanted by a “CC team” consisting of physicians and nurses trained in the technique of catheter insertion. All devices were inserted in the procedure room using the ultrasound-guided technique, with strict aseptic conditions and maximum barrier precautions. Patients with indwelling devices were screened on admission for tunnel and catheter infections prior to initiation of the treatment regimen. 

For PICC implantation, peripheral veins of the non-dominant arm (basilic or brachial veins) were identified and cannulated under local anesthesia without sedation. The brachial vein was typically the vessel of choice due to its diameter unless other anatomical alterations occurred. For CICC insertion, the right subclavian vein was usually the vessel of choice due to lower catheter-related infections in this site. A chest X-ray was routinely performed in all patients to verify the correct placement of the tip (close to the cavoatrial junction). All catheters were attached to the skin with sutureless devices (StatLock, BD, Franklin Lakes, NJ, USA). Types of implanted CICCs have been standardized—non-tunneled 7 Fr triple lumen catheter. Most CICCs were implanted into the subclavian vein, but only 4 patients had CICCs implanted into the jugular vein due to anatomical conditions. Most PICCs were 5 Fr, and only 3 patients had different sizes of the catheter: one triple lumen 7 Fr and 2 double lumen 4 Fr. The size of the PICC depended on the diameter of the vein and the catheter/vein diameter ratio. There were no serious insertion complications in either group. Minor complications included catheter tip malposition in 1 patient in the PICC group and a small hematoma in 1 patient in the CICC group. All patients in both groups underwent high-dose conditioning chemotherapy followed by stem cell infusion, including 44 patients receiving high-dose melphalan (MEL200/MEL140; Melphalan, Zentiva, Prague, Czech Republic), one patient receiving busulfan/melphalan (BuMel; Busilvex, Pierre Fabre Medicament, Boluogne Billancourt Cedex, France; Melphalan, Zentiva, Prague, Czech Republic), one patient receiving fludarabine/busulfan (FluBu; Fludarabine, Accord Healthcare, Warsaw, Poland; Busilvex, Pierre Fabre Medicament, Boluogne Billancourt Cedex, France;), and two patients receiving carboplatin/etoposide (CE; Carboplatin-Ebewe, EBEWE Pharma, Unterach, Austria; Etoposid-Ebewe, EBEWE Pharma, Unterach, Austria) conditioning. Subsequently, patients in both groups received cryopreserved stem cells via gravity infusion with no infusion rate enhancement. Patients were monitored for signs of hematopoietic recovery and, upon fulfilling generally accepted engraftment criteria, discharged to outpatient care. All CICCs were removed after the completion of therapy. Most PICCs were also removed, with the exception of 2 GCT patients who underwent 3 cycles of high-dose therapy on a single PICC.

### 2.2. Statistical Analysis

The two-tailed Mann–Whitney U test was used to analyze the differences between the measurement variables. Principal component analysis (PCA) was used in order to reveal any particular pattern within our dataset. Quantitative variables are expressed as mean, standard deviation (SD), and min–max range (range). All calculations were performed in Rstudio (2022.12.0). *p* value < 0.05 is considered statistically significant.

## 3. Results

Table 2 depicts the results of the comparison of the PICC and CICC groups. The mean age was 53.85 and 59.03 years for the PICC and CICC groups, respectively, with no statistical differences between the groups (*p* = 0.09). The total CD34^+^ dose was significantly different between the groups (*p* < 0.001). The mean CD34^+^ dose in the PICC group was 8.61 million CD34^+^ cells/kg, while in the CICC group, it was 12.67 million CD34+ cells/kg. There was no significant difference between the mean CD34^+^ volume in the CICC group (213.25 mL) and the CICC group (170.94 mL). The mean length of CD34^+^ infusion was longer in the PICC group (32.8 min) than in the CICC group (7.58 min), and this difference was statistically significant (*p* < 0.001). Similarly, the mean infusion velocity significantly differed between the groups (*p* < 0.001) and was 7.78 mL/min and 16.53 mL/min in the PICC and CICC groups, respectively.

Prolonged exposure of CD34^+^ cells to DMSO, due to the lower infusion velocity and longer infusion time in the PICC group, had no effect on the transplantation procedure. Engraftment times and the length of stay are efficacy indicators of stem cell transplantation. The mean time of neutrophil engraftment in the PICC group was 11.2 days, while in the CICC group, it was 11.67 days, and the difference was not statistically significant (*p* = 0.25). Simultaneously, the mean time of platelet engraftment was similar in both groups (*p* = 0.85) and equaled 13.16 days and 13.94 days in the PICC and CICC groups, respectively. The mean length of hospitalization was in both groups alike (*p* = 0.39). Patients with PICC spent approximately 22.05 days in the hospital, while patients with CICC spent 21.36 days.

**Figure 1:**
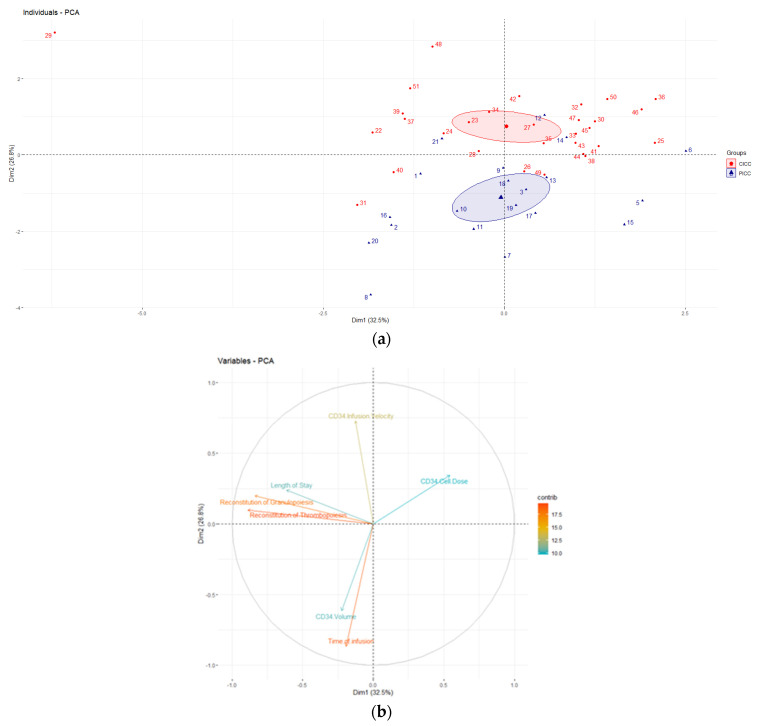
Principal component analysis (PCA) depicting the analyzed dataset. (**a**) Graph of individuals; (**b**) graph of variables.

PCA is a statistical technique that enables the analysis of a dataset as a whole and detects associations that are not visible when the analysis is limited to a simple comparison between groups. Analysis (Figure 1) revealed that the PICC and CICC groups separate from each other forming two different clusters. The first two principal components contribute to 59.3% of the variability in the analyzed sample. Time of infusion, reconstitution of granulopoiesis, and thrombopoiesis appear to have the greatest influence on the principal component. Moreover, vectors representing the time of infusion and infusion velocity form an approximately 90° angle, with vectors depicting the reconstitution of granulopoiesis, thrombopoiesis, and length of stay. This suggests that the variables of the two groups do not correlate with each other, i.e., the clinical outcome of transplantation, exemplified by platelet and neutrophil engraftment, and length of stay are not affected by prolonged infusion time and low infusion velocity, despite the fact that the Mann–Whitney U test revealed that time of infusion and infusion velocity significantly differed between the PICC and CICC groups. Although PCA did not reveal any additional relationships in our study, this supports the initial analysis and depicts that the PICC and CICC groups are truly different and can be distinguished by time of infusion and infusion velocity.

## 4. Discussion

CVCs are essential vascular access devices for the safe management of patients undergoing stem cell transplantation. The multipurpose central line facilitates the infusion of physically incompatible drugs, cytostatics, blood-derived products, anti-infectives, vasopressors, and highly osmotic nutritional solutions. CICCs are usually the catheters of choice due to their widespread availability and the preference of most operators. Unfortunately, CICCs generate specific problems with both implantation and removal. The most common complications involve arterial puncture, nerve damage, arterial catheterization, tissue hematoma, hemothorax, air embolism, and pneumothorax, which may have a negative impact on the outcome of the transplant procedure. McGee et al. reported that CICC implantation is associated with mechanical complications in 6–19% of cases, with pneumothorax as high as 3% [14]. Ultrasound guidance during insertion tends to reduce the incidence of serious adverse events. In a prospective, multicenter cohort study of over 12,000 ultrasound-guided CVCs, Adrian et al. reported that minor mechanical complications occurred in 8% of insertions and major complications in less than 1%, with pneumothorax being the most frequent one [15]. On the contrary, the implantation of PICC is safe and devoid of the abovementioned hemothorax and pneumothorax. Studies by Bellesi and Sakai showed that PICC was associated with fewer serious insertion- and removal-related complications than CICC [16,17]. No major insertion-related complications were observed, and local hematoma was the most common minor complication (1.6%).

Other concerns with the use of PICC include the risk of central line-associated bloodstream infections (CLA-BSI) and catheter-related thrombosis (CRT). Bellesi et al. evaluated the safety of PICC and the risk of CLA-BSI in 57 patients undergoing high-dose therapy. The risk was determined as low, reaching 3.3% (2/60), which corresponds to 1.5 CLA-BSI per 1000 PICC days [4]. A systematic review of published prospective studies by Maki et al. suggests that in high-risk patients, defined as stem cell transplant recipients or leukemia patients, the risk of CLA-BSI is slightly lower in PICC than in standard CICC (2.1 CLA-BSI per 1000 PICC days vs. 2.7 CLA-BSI per 1000 CICC days). Nakaya et al. compared the risk of CLA-BSI between 472 CICC and 557 PICC in adult patients with hematological diseases and demonstrated that PICC was independently associated with a reduced risk of CLA-BSI [5.11 and 3.29 per 1000 catheter days in CICC and PICC groups, respectively (*p* = 0.024)] [18].

Regarding CRT, various reviews have presented conflicting results in recent years. A systematic review and meta-analysis concluded that PICCs are associated with an increased risk of CRT compared to other tunneled CVCs but not pulmonary embolism [19]. It is worth mentioning that most of the published data derive from oncologic patients, and stem cell transplantation may have varying degrees of CRT risk, especially in the case of severe thrombocytopenia, which is common in the transplantation setting. Morano and Frachiolla reported a low incidence of PICC-associated CRT in hematological patients, of 2 to 8%, comparable to the rate of conventional central line-associated thrombosis [20,21].

Interestingly, despite the low CLA-BSI and acceptable CRT rate, which are the two most important complications in the transplantation setting, PICCs are rarely used for the infusion of cryopreserved stem cells. The rationale for this practice is not based on evidence, but the general hypothesis is that prolonged infusion of stem cells causes damage to human progenitor cells (HPCs) through excessive exposure to DMSO. Standard PICCs have a small caliber (usually 4 to 5 Fr) and an increased length (average 30 to 50 cm) compared to CICCs and therefore have high flow resistance. Depending on the caliber, the PICC can achieve a flow rate of only 2–4 mL/min with gravity infusion [22], which extends the HPC infusion beyond the recommended 20 min/bag [23]. The rate of infusion can be significantly increased with infusion pumps, but unfortunately, pumps are not used to administer HPC due to the paradigm that the pressure from the pump can cause HPC barotrauma [24]. Because of these assumptions and the lack of evidence-based consensus, most transplant units employ gravity infusion, which excludes PICC as a reliable access to HPC infusion. Interestingly, Kissoon and colleagues performed a retrospective analysis of 114 pediatric patients who received HPC via an intravenous injection pump. The data obtained support this method of HPC administration as no graft failure or poor graft function has been reported. Furthermore, the engraftment kinetics of both myeloid and platelet lineages were comparable to historical data from obtained from National Marrow Donor Program (NMDP) [25].

The cytotoxicity of DMSO has been extensively investigated in numerous studies that have examined its effects exerted on various cellular subpopulations. The results of these studies appear to provide background for the assumption that a prolonged infusion rate would have a negative impact on the viability of CD34^+^ stem cells. De Abreu Costa and colleagues demonstrated that exposure to 1% and 2% DMSO reduced lymphocyte proliferation. In addition, the results indicated that 20% DMSO was able to induce hemolysis of peripheral blood mononuclear cells [26]. Liseth et al. conducted an interesting study in which they investigated the survival of CD34^+^ cells when the PBSCs used for autologous transplantation were cryopreserved in various concentrations of DMSO. In their research, PBSCs were frozen in 2%, 4%, 5%, and 10% DMSO solutions. The results obtained showed no significant differences in CD34^+^ cell viability when the cells were cryopreserved with 4% or 5% DMSO. Nevertheless, when cells were cryopreserved in either lower or higher DMSO concentrations, their viability markedly decreased [27]. Yi and co-workers explored the dose-related effects of DMSO on red blood cells, platelets, and vascular endothelial cells in vitro. They demonstrated that even low DMSO concentrations were able to induce hemolysis and impair platelet aggregation. Moreover, it inhibited proliferation and increased apoptosis of vascular endothelial cells [28]. The abovementioned studies focused on the dose rather than the exposure time. However, it appears that DMSO-related cytotoxicity is both dose- and time-dependent. Fry et al. demonstrated that 7.5–10% is the optimal concentration of DMSO, and the maximum exposure time should be limited to <1 h prior to freezing and 30 min after thawing. It should be noted that in their study, hematopoietic progenitor cells were derived from umbilical cord blood [29]. On the other hand, data regarding DMSO toxicity to HPCs and supporting high flow rates during infusion are ambiguous. Rowley and Branch indicate that longer than recommended exposure has no effect on stem cell viability and that DMSO was not found to be toxic to HPCs at concentrations of 5% or 10% at either 4 °C or 37 °C for incubation times up to 1 h after thawing [23,30].

These findings suggest that prolonged infusions of HPCs through small catheters are feasible and safe. Although the use of PICCs is broadly explored in the conventional therapy of oncohematology patients, they are rarely utilized in stem cell transplantation. Recently, Bellesi et al. [16] prospectively evaluated the safety and efficacy of 57 PICCs inserted into autologous PBSC recipients. They demonstrated almost no insertion-related complications and a low rate of CLA-BSI and CRT, reaching 3.3% and 5.0%, respectively. No primary graft failure was reported. Similarly, Mariggio et al. reported a low CRT rate among allogeneic PBSC recipients, with CLA-BSI higher than previously reported but dependent on PICC type [31]. Choon et al. reported similar neutrophil and thrombocyte engraftment in a small group of patients receiving autologous stem cells via PICC, suggesting that the reduced infusion rate had no impact on engraftment and the overall outcome of the procedure [32]. Similarly, our data indicate that PICCs are safe and effective for the infusion of cryopreserved stem cells, with no noticeable detrimental effect of prolonged infusion on HPCs. The mean infusion rate in our study was 32.8 min, and the longest PBSC infusion lasted 84 min. Although the cytotoxicity of DMSO appears to be dose- and time-dependent, the exposure time during PICC infusion appears to be insufficient to induce cytotoxic effects. Furthermore, PICCs have potential advantages over CICCs, as they are more comfortable for patients, have a decreased infection risk, have a low rate of implantation complications, and allow for long outpatient care and an additional transplantation procedure if required. The issue of CRT in the context of PICC utilization in high-risk hematological patients requires further investigation, as there are factors that may be considered protective in this specific patient population, such as thrombocytopenia.

Our study provided interesting results and supported the implementation of PICCs in stem cell transplantation settings. However, it has some minor limitations. First of all, its retrospective character should be mentioned. Second, due to the fact that the use of the PICCs was recently implemented in our facility, the number of patients analyzed was relatively small. Therefore, we firmly believe that in the upcoming future, our results should be explored in prospective studies with bigger sample sizes. This would additionally support the widespread implementation of PICCs in the transplantation of cryopreserved PBSCs.

## 5. Conclusions

Our study showed that the use of PICC is safe and effective for the infusion of cryopreserved stem cells compared to conventionally inserted large central venous catheters. Despite a statistically significant reduction in the infusion rate and thus a longer infusion of total PBSC, we did not observe any differences in neutrophil and platelet reconstitution between the two groups. Interestingly, the total number of CD34^+^ progenitor cells was lower in the PICC group. We experienced no significant catheter-related complications. The length of hospitalization did not differ between the groups, suggesting similar outcomes of transplant procedures despite differences in infusion rates.

## Figures and Tables

**Table 1:** Characteristics of the study group.

	Patients n = 49 100(%)	
Catheter type	PICC	CICC
Male/Female	8/8	16/17
Median Age, years	56 (35–65)	59 (29–71)
Type of disease		
PCM	11 (69%)	33 (100%)
AL	1 (6%)	0 (0%)
GCT	3 (19%)	0 (0%)
AML	1 (6%)	0 (0%)
Type of HSCT		
Allo-HSCT	1 (5%)	0 (0%)
Auto-HSCT	20 (95%)	33 (100%)
Number of HSCT	total = 21 (100%)	total = 33 (100%)
Single	13 (81%)	33 (100%)
Double	1 (6%)	0 (0%)
Triple	2 (13%)	0 (0%)
Conditioning regimen		
MEL200	11 (52%)	30 (91%)
MEL140	2 (10%)	2 (6%)
CE	7 (33%)	0 (0%)
FluBlu4	1 (5%)	0 (0%)
BuMel	0 (0%)	1 (3%)
Catheter size		
Fr4	3 (18%)	0 (0%)
Fr5	16 (70%)	0 (0%)
Fr6	2 (12%)	0 (0%)
Fr7	0 (0%)	33 (100%)
Number of lumens		
1	3 (18%)	0 (0%)
2	16 (70%)	0 (0%)
3	2 (12%)	33 (100%)
Insertion site		
Right subclavian vein	0 (0%)	85%
Left subclavian vein	0 (0%)	3%
Right jugular vein	0 (0%)	12%
Left basilic vein	15 (68%)	0 (0%)
Right basilic vein	6 (32%)	0 (0%)

Patients and catheter characteristics. PCM—plasma cell myeloma, AL—light chain amyloidosis, AML—acute myeloid leukemia, GCS—germ cell tumor, Allo-SCT—allogeneic stem cell transplantation, ASCT—autologous stem cell transplantation, MEL—melphalan, CE—carboplatin/etoposide, FluBu4—fludarabine/busulfan, BuMel—busulfan/melphalan, Fr—French gauge (1 Fr = 1/3 mm).

**Table 2:** Comparison between the PICC and CICC groups.

Parameter	PICC Mean ± SD; (Range)	CICC Mean ± SD; (Range)	*p* Value
Age	53.85 ± 11.36; (35–69)	59.03 ± 8.83; (29–71)	0.09
CD34 cell dose (×10^6^/kg)	8.61 ± 10.26; (2.15–22.1)	12.67 ± 9.56; (1.9–42.4)	<0.001
CD34 volume (mL)	213.25 ± 110.19; (37–498)	170.94 ± 83.99; (60–406)	0.13
Length of infusion (min)	32.8 ± 19.94; (6–84)	7.58 ± 3.69; (3–19)	<0.001
Infusion velocity (mL/min)	7.78 ± 4.04; (2.69–16.0)	16.53 ± 6.88; (11.83–45)	<0.001
Neutrophil engraftment (day)	11.2 ± 0.95; (10–18)	11.67 ± 0.89; (11–15)	0.25
Platelet engraftment (day)	13.16 ± 2.63; (10–18)	13.94 ± 3.73; (10–27)	0.85
Length of hospitalization (days)	22.05 ± 3.12; (18–38)	21.36 ± 4.08; (17–38)	0.39

Neutrophil engraftment—first of 3 consecutive days with an absolute neutrophil count of >500/μL in the post-transplantation period. Platelet recovery—first of 3 consecutive days with a platelet count of 20,000/μL or higher in the absence of platelet transfusion for 7 consecutive days. Mann–Whitney U Test, *p* value < 0.05 is considered statistically significant.

## Data Availability

The data are available on request from the corresponding author.
